# Use of Physician-in-Triage Model in the Management of Abdominal Pain in an Emergency Department Observation Unit

**DOI:** 10.5811/westjem.2016.10.32042

**Published:** 2017-01-19

**Authors:** John R. Marshall, Robert Katzer, Shahram Lotfipour, Bharath Chakravarthy, Siri Shastry, Jessica Andrusaitis, Craig L. Anderson, Erik D. Barton

**Affiliations:** University of California, Department of Emergency Medicine, Irvine, California

## Abstract

**Introduction:**

Given the nationwide increase in emergency department (ED) visits it is of paramount importance for hospitals to find efficient ways to manage patient flow. The purpose of this study was to determine whether there is a significant difference in success rates, length of stay (LOS), and other demographic factors in two cohorts of patients admitted directly to an ED observation unit (EDOU) under an abdominal pain protocol by a physician in triage (bypassing the main ED) versus those admitted via the traditional pathway (evaluated and treated in the main ED prior to EDOU admission).

**Methods:**

This was a retrospective cohort study of patients admitted to a protocol-driven EDOU with a diagnosis of abdominal pain in a single university hospital center ED. We obtained compiled data for all patients admitted to the EDOU with a diagnosis of abdominal pain that met EDOU protocol admission criteria. We divided data for each cohort into age, gender, payer status, and LOS. The data were then analyzed to assess any significant differences between the cohorts.

**Results:**

A total of 327 patients were eligible for this study (85 triage group, 242 main ED group). The total success rate was 90.8% (n=297) and failure rate was 9.2% (n=30). We observed no significant differences in success rates between those dispositioned to the EDOU by triage physicians (90.6%) and those via the traditional route (90.5 % p) = 0.98. However, we found a significant difference between the two groups regarding total LOS with significantly shorter main ED times and EDOU times among patients sent to the EDOU by the physician-in-triage group (p< .001).

**Conclusion:**

There were no significant differences in EDOU disposition outcomes in patients admitted to an EDOU by a physician-in-triage or via the traditional route. However, there were statistically significant shorter LOSs in patients admitted to the EDOU by triage physicians. The data from this study support the implementation of a physician-in-triage model in combination with the EDOU in improving efficiency in the treatment of abdominal pain. This knowledge may spur action to cut healthcare costs and improve patient flow and timely decision-making in hospitals with EDOUs.

## INTRODUCTION

In a 2015 American College of Emergency Physicians (ACEP) poll, 75% of surveyed emergency physicians felt that patient volume had increased since implementation of the Affordable Care Act (ACA) in 2014.^1^ To address this, many emergency departments (ED) have formed observation units (EDOU) in an effort to expeditiously manage patients with an expected length of stay (LOS) less than 24 hours. EDOUs have been shown to reduce healthcare costs via reduction in both initial hospital admissions as well as overall patient hospital LOS.^2^–^7^ One recent study showed a mean annual cost savings of $108 million from avoidance of 235,000 hospital admissions for patients presenting to the ED with a chief complaint of syncope.^3^ A 2012 study also projected that a nationwide adoption of EDOUs would lead to a projected annual cost savings of $3.1 billion.^7^

In an effort to further improve throughput and decrease patient LOS, some hospitals have implemented physician-in-triage models in which an EP evaluates, treats and at times dispositions patients in the triage area, bypassing the main ED. The physician-in-triage model has also been shown to decrease patient LOS within the ED.^8^,^9^

Studies have shown that highly subjective conditions such as abdominal pain are potentially difficult to manage in EDOU settings.^10^–^12^ Abdominal pain is also the most common chief complaint among ED visits, comprising 8% of total visits in the most recent available National Hospital Ambulatory Medical Care survey in 2011.^11^ The average failure rate among EDOUs on a national level is between 20–30%. If admission rates far exceed this percentile, suspension of the hospitals EDOU privileges may occur.^13^

Increased usage of both EDOUs and a physician-in-triage model may lead to a significant number of patients with chief complaints of abdominal pain being admitted to the EDOU by a triage physician. There is currently limited literature on clinical outcomes and the relative success rates of patients admitted to the EDOU by a triage physician versus those admitted via the main ED.

The purpose of this study was to look at patients admitted to the EDOU with a diagnosis of undifferentiated abdominal pain to determine whether there is a significant difference in success rates (disposition home) of these patients admitted directly to the EDOU by a triage physician (bypassing the main ED) versus those admitted via the traditional pathway (evaluated and treated in the main ED prior to EDOU admission). Our study additionally sought to examine the effects of gender, age, and insurance payer status on success rates. We also examined the impact of physician-in-triage evaluation and subsequent EDOU admission on patient LOS.

## METHODS

This was a retrospective cohort study of 327 patients admitted to the EDOU under an abdominal pain protocol from July 1, 2015, to January 14, 2016, in a single university hospital center ED. We obtained institutional review board approval prior to data extraction and analysis. The total population was divided into two cohorts, those dispositioned to the EDOU by an attending physician working in triage and those dispositioned by an attending physician in the main ED.

We determined that the sample size necessary to obtain significant results in the study was 61. This was calculated using a 95% confidence interval, 10% margin of error, response distribution of 26% (85/237) and our known population size 327. Response distribution was 26% (85/327). Our sample achieved was 85 patients.

**Figure 1:**
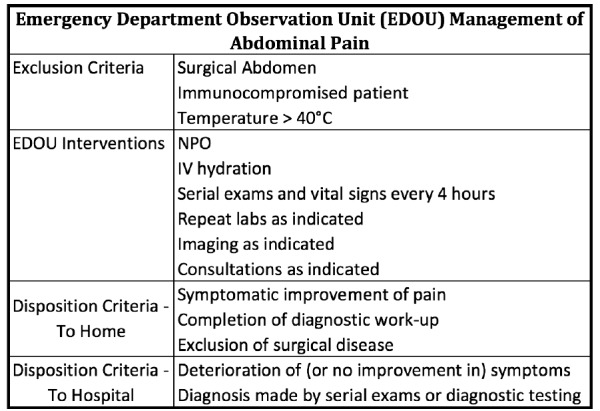
Emergency department observation unit (EDOU) management of abdominal pain.

All patients admitted to the EDOU under the EDOU protocol of abdominal pain who met the departmentally set criteria were included in this study (Figure 1). The EDOU abdominal pain protocol includes strict exclusion criteria, interventions, disposition criteria, and a timeframe. The exclusion criteria are surgical abdomen, immunocompromised status, and a fever of >103F. Interventions per the protocol are NPO, intravenous hydration, serial exams and vital signs every four hours. Imaging (radiograph, computed tomography and ultrasound), consultations, and repeat labs are all decided by both main ED and triage physicians as indicated. The protocol establishes the criteria for disposition to home as improvement of pain, completion of diagnostic work up, and exclusion of surgical disease. The criteria for admission to the hospital are deterioration or no improvement, or diagnosis established. Lastly, the protocol establishes the time frame for treatment as 6–23 hours.

The EDOU is run primarily by nurse practitioners with attending EPs in the main ED and triage area available if clinical questions arise or a change in patient status arises. The protocol is decided by the treating EP and the plan and presumed course is discussed in detail on sign out to the nurse practitioner. Prior to the initiation of the EDOU, all clinicians were provided with training that included information on EDOU operations, step-by-step instructions on how to admit patients to the EDOU, and copies of the current protocols. This training was provided via live faculty meetings and email. All current protocols are available in several locations in the main ED, electronic medical record, and in the EDOU. Variation from the protocol is rare but can occur when a clinician deems it necessary.

The physician in triage at the study site evaluates patients within the triage area between 10 a.m. and 1 a.m. The physician in triage is a board certified/board eligible EP and clinical instructor. Staff members who work as the physician in triage also work in the main ED, and triage shifts comprise a portion of each faculty member/fellow’s monthly clinical shift requirement.

We obtained compiled data for all patients admitted to the EDOU with a diagnosis of undifferentiated abdominal pain who met EDOU admission criteria, along with whether the admission outcome was a success or failure. We further stratified the data for each cohort gender (male or female), age group (16–40, 41–60, 61–100), and payer status (self, private, Medi-Cal, Medicare, VA). Data on length of main ED time, EDOU time, and total time were also collected. We analyzed the success rates, LOS, and subgroup data for each cohort using STATA analytical software for significant differences using a two-sample t-test. To assess for significant findings in the overall success rates, multiple groups were compared using chi-square and Fisher’s exact tests.

## RESULTS

**Figure 2:**
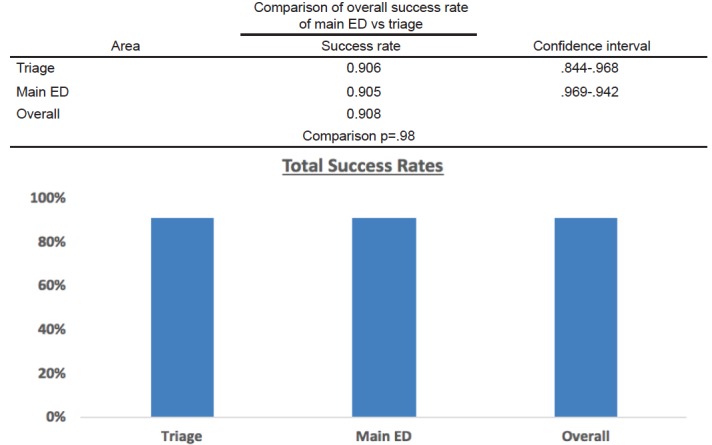
Comparison of overall success rate (discharge home within 24 hours) of main ED vs triage.

A total of 327 patients admitted to the EDOU with a diagnosis of abdominal pain were eligible for this study. Of these, 85 were seen by triage physicians and 242 were seen via the traditional route in the main ED. Overall, the total success rate was 90.8% (n=297) and failure rate was 9.2% (n= 30) (Figure 2). The largest percentage of patients grouped by gender, age, and payer status were female 63% (n=187), age range of 16–40- 50.5% (n=150), and Medi-Cal 54.5% (n= 162), respectively. The oldest patient included in the study was 90 and the youngest was 16.

**Figure 3:**
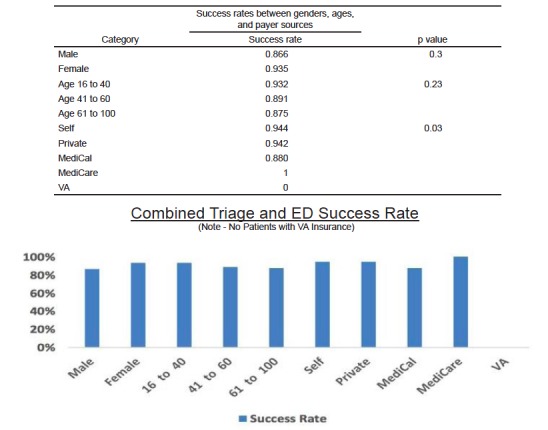
Success rates (discharge home within 24 hours) between genders, ages, and payer sources. *VA,* Veterans Administration.

**Figure 4:**
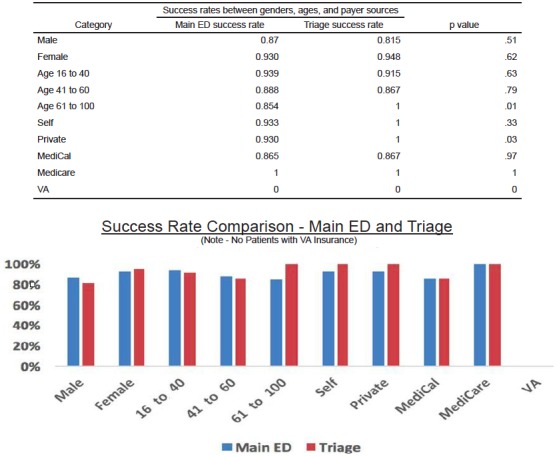
Comparison of success rates (discharge home within 24 hours) of ages, gender, and payer sources betwen main ED and triage. *VA,* Veterans Administration.

When comparing between the two cohorts, we observed no significant differences in success rates between those dispositioned to the EDOU by triage physicians (90.6%) and via the traditional route (90.5 % p = 0.98) (Figure 2). In looking at the total population, we observed significant differences among groups only regarding gender p=0.03 and payer status p=0.03 when a chi-square test and Fisher’s exact test were used to compare subgroups (Figure 3). When comparing the subgroups among the cohorts, statistically significant differences were found in the private pay groups and the 61–100 age group (Figure 4).

**Figure 5:**
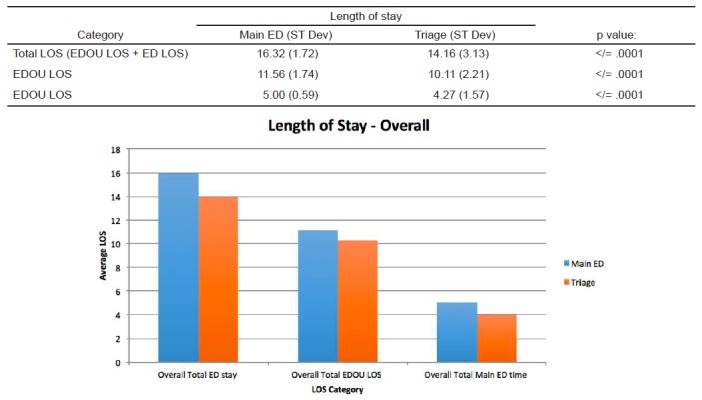
Data comparing length of stay (LOS) data in main emergeny department (in hours) vs emergency department observation unit (EDOU).

We used a t-test of times assuming unequal variances to analyze any significant differences in overall total stay (main ED time + EDOU time), EDOU time, and main ED time. The mean times for the total stay, EDOU time, and main ED time were 16.32 hours, 11.56 hours, and 5.00 hours respectively for the main ED and 14.16, 10.11, 4.27 for the triage group respectively. In all three categories we found significant difference in times with the patients who were sent to the EDOU directly from triage versus those sent from the main ED p< .001 (Figure 5). Given that the mean total LOS for the EDOU from triage group was two hours less than the traditional group, a total of more than 170 bed hours were saved by admitting that group straight from triage over a period of six and a half months.

## DISCUSSION

As the number of EDOUs nationwide increases, there will be a growing need to safely use them to positively impact patient care and improve allocation of hospital resources. The primary purpose of this study was to examine whether the physician-in-triage model could be safely applied in dispositioning patients with a highly subjective and difficult-to-manage complaint to the EDOU. Also, we aimed to unmask any significant differences in time and resources saved as well as assess any significant differences in gender, age, and other demographic data that may have existed between the cohorts.

In this study, there were no significant differences in the EDOU disposition outcomes in patients admitted to an EDOU from triage or via the traditional route. Additionally, we also discovered a significant difference in LOS between the two cohorts of patients admitted from triage versus those admitted from the main ED. The triage patients in fact had shorter lengths of stays in each phase of their hospitalization: total stay, EDOU time, and main ED time. These two findings of equal success achieved with shorter lengths of stay, suggest that the EP’s clinical intuition of assessing highly subjective complaints such as abdominal pain can be relied upon to make rapid EDOU disposition decisions for our patients. This can have a significant impact on patient flow through the ED, in turn having significant impact on resource allocation, efficiency, costs, and even patient satisfaction.

There is a growing body of research regarding the innovation of the EDOU. Currently, however, this research is focused on the EDOU and looks at operation designs within the units themselves such as protocols and success rates, and does not address the interplay between the physician-in-triage model with EDOUs. As both the physician-in-triage model and the EDOU are fairly new system designs in emergency medicine that are gaining in popularity but have yet to garner unanimous and ubiquitous support, this study adds support to the implantation of both systems, the physician-in-triage model and the EDOU, to further decrease LOS in patients with abdominal pain.^2^–^7^

Our findings of equal EDOU success rates between triage patients and main ED patients was surprising. Traditionally, it has been thought that more accurate disposition decisions would have been achieved after a thorough workup in the main ED rather than through a brief triage assessment. However, this study showed that triage physicians, even with their limited time with the patient and lack of objective data, are able to make equivalent disposition decisions. This would suggest that perhaps the physician’s clinical gestalt is highly sufficient in making quick disposition decisions.

We also found that patients admitted to the EDOU from triage had a shorter LOS then those from the main ED. Several explanations are proposed for this. Presumably, the triage-to-EDOU path is more efficient and less time is spent with the patient simply waiting for a main ED bed to open. Perhaps the triage group received medications sooner because they were quicker to get to providers who had time to administer medications. Or perhaps the patients in the triage group were able to receive stronger medications not available to their main ED cohorts because those patients were still in the waiting room. Many similar mechanisms could be proposed. Regardless of the underlying reason, the increase in patient flow and efficiency is an undeniable improvement.

When considering the influence of a patient’s insurance on outcome, this study showed a statistically significant improved success rate of triage versus main ED in the private-pay group. One reason for this difference may be the fact that patients in the private-pay group likely have more reliable follow-up options in place and easier access to primary care/specialty follow up, allowing for a quicker and easier discharge. The other-payer groups did not have any differences in outcome.

## LIMITATIONS

Potential limitations of this study include its size and patient population. The control group was much larger than the study group (85 from triage versus 242 from the main ED) because in practice it is more common for a triage physician to quickly see a patient and send the patient to the main ED for a more complete evaluation, workup, and decision, than for the triage physician to admit the patient directly to the EDOU. Those patients who are briefly seen by triage are only counted in the control group, not in the triage group because they were not *admitted* from triage. With only 327 total patients, 85 of whom were admitted from triage, this is still a relatively small study and it should be acknowledged that with small sample sizes data obtained may be less valid. It is highly likely that the significant findings regarding age, gender, and payer status may be explained due to small populations within each group.

The specific patient population for this study should also be considered. Our most represented patient group was females aged 16–40 with Medi-Cal insurance, which is most likely not representative of the entire population. It is important to consider whether this skew in patient population could have altered our findings and, importantly, whether our findings would be pertinent to a facility that did not share similar population characteristics. We also recognize that this study looks solely at one protocol and findings may vary significantly depending on protocol. Clinicians from triage and the main ED used imaging as was indicated, but this study did not collect data on the frequency or type of imaging used between the groups of clinicians.

Additionally, because triage physicians are only evaluating and dispositioning patients between the hours of the 10 a.m. and 1 a.m., the results of our study may be subject to an element of selection bias. Our study did not specifically examine data on variation in acuity or change in EDOU success rates based upon presentation during the hours within which no physician in triage is present. While our study did examine total lengths of stay as an outcome measure, we did not examine the specific time of placement within the EDOU/time of discharge and the influence of this measure on total LOS. Future studies may benefit from only examining patients admitted to the EDOU between 10 a.m. and 1 a.m.with presentation between 1a.m. and 10 a.m. acting as an exclusion criteria. Future studies may also benefit from examining specific time of placement in and discharge from the EDOU to determine whether a greater proportion of patients are discharged from the EDOU at certain times and if, accordingly, placement within the EDOU at particular times influences LOS within either group. This also may represent a future direction of study given that patient satisfaction is an important quality measure that is being increasingly emphasized nationwide. Lastly, we acknowledge that some of the EDOU lengths of stay included main ED boarding time as they awaited bed availability in the EDOU.

## CONCLUSION

The data from this study serve to support that the use of the physician-in-triage model in combination with the EDOU can improve ED efficiency and, most importantly, safely treat a highly subjective complaint such as abdominal pain. This finding will likely have beneficial effects on patient flow, cutting departmental costs, and improving patient satisfaction. Given the prevalence of abdominal pain complaints as well as the potential cost savings associated with successful use of the EDOU and decreased patient LOS through use of the physician-in-triage model, there is a significant need for further investigation on this topic and for identification of factors leading to or detracting from increased success rates. Future studies should also aim to look at other EDOU protocols to see if similar conclusions can be drawn. As continued support for EDOUs is often predicated upon maintaining a low failure rate, it is of paramount importance that predictors of EDOU success/failure be investigated in order to better predict successful disposition at time of admission to the EDOU.
